# Remote interventions to improve exercise behaviour in sedentary people living with and beyond cancer: a systematic review and meta-analysis

**DOI:** 10.1186/s12885-021-07989-0

**Published:** 2021-03-24

**Authors:** Saïd Ibeggazene, Rebecca Turner, Derek Rosario, Liam Bourke

**Affiliations:** 1 College of Health, Wellbeing and Life Sciences, Sheffield Hallam University, Sheffield, UK; 2 Department of Oncology and Metabolism, University of Sheffield, Sheffield, UK

**Keywords:** Cancer survivorship, Physical activity, Exercise behaviour, Telerehabilitation, Systematic review, Meta-analysis

## Abstract

**Background:**

The COVID-19 pandemic has forced many cancer services to consider a transition to a remote format of delivery that is largely untested. Accordingly, we sought to perform a systematic review of the effects of remotely delivered interventions to improve exercise behaviour in sedentary adults living with and beyond cancer.

**Methods:**

Eligible studies were randomised controlled trials comparing a remotely delivered exercise intervention to a usual care comparison in sedentary people over 18 years old with a primary cancer diagnosis. Nine electronic databases were searched from inception to November 2020.

**Results:**

The review included three trials, totalling 186 participants. Two of the included trials incorporated prescriptions that meet current aerobic exercise recommendations, one of which also meets the guidelines for resistance exercise. No trials reported an intervention adherence of 75% or more for a set prescription that meets current exercise guidelines.

**Conclusion:**

There is little evidence suggesting that remote exercise interventions promote exercise behaviours or improve physical function in sedentary adults living with and beyond cancer. The development and evaluation of novel remote exercise interventions is needed to establish their usefulness for clinical practice. Given the social response to the COVID-19 pandemic, further research in this area is urgently needed.

**Supplementary Information:**

The online version contains supplementary material available at 10.1186/s12885-021-07989-0.

## Background

Regular exercise can benefit the lives of people living with and beyond cancer. Despite this, only 13–40% cancer survivors are physically active [1–4]. Supervised rather than unsupervised exercise is more effective at promoting physical activity in previously inactive individuals [5], and recognised as an important component of cancer care by healthcare organisations in several developed countries [6–8]. Widespread provision of supervised exercise services for cancer is not well established.

As of early 2020, delivery and introduction of many direct face-to-face health services have been stymied by the COVID-19 pandemic across the UK [9–11] and other afflicted nations [12–15] which has caused many providers to seek alternative modes of service delivery. This is an appropriate initial response as the risks from contracting COVID-19 to cancer populations are higher than in the general population, principally due to advanced age and the presence of comorbidities [16]. Exercise facilities were associated with some early COVID-19 clusters [17, 18]. In some countries exercise facilities have reopened. Through the introduction of protective measures and behavioural reactions to the COVID-19 pandemic, the risks associated with attending exercise facilities may have reduced, however empirical data to support this is lacking. If, and in what form, face-to-face exercise programmes for cancer populations recommence is at present uncertain. Considering this, interventions that deliver exercise interventions remotely, such as in an individual’s home environment, may become an essential tool for promoting exercise in people with cancer.

Telemedicine is anticipated to play an increasing role in healthcare [19, 20] and is being rapidly adopted by healthcare services [21], which may extend to clinical exercise services for people with cancer in the future. The application of remotely delivered exercise interventions may also have the advantages of enhanced reach and accessibility by overcoming known barriers to participation in face-to-face supervised exercise. These physical and psychological barriers include: poor or cold weather, lack of time [1, 22, 23], distance to travel to facility [24], negative perceptions of programme and transport costs [25, 26], inconvenient timings of exercise programmes [25, 27]. Furthermore, cancer patients frequently state a preference to exercise in their home environment and surrounding areas [23, 25, 27, 28] though this is not always the case [29].

Exercise interventions are often lauded to have substantial potential for cost savings to healthcare systems [30], however, at present there is mixed evidence for the cost-effectiveness of these in cancer populations [31, 32]. Remote delivery of exercise interventions could potentially circumvent some of the costs of exercise services related to upkeep of facilities and specialised equipment, whilst offering a scalable service that caters to individuals beyond a limited urban locale. Despite this promise, the effectiveness of remotely delivered exercise interventions in people with cancer is unclear. Previous systematic reviews of remote physical activity interventions in cancer survivors have been unable to provide an answer as to whether remotely delivered exercise interventions are likely to be effective to promote exercise behaviours or influence health outcomes [33, 34]. Potential reasons for the ambiguous conclusions of these reviews include methodological limitations of the included studies, heterogenous intervention designs, inadequate control conditions and inclusion of participants who are already active at baseline. Therefore, the purpose of this review is to assess the most recent evidence of the effects of remote exercise interventions in people living with and beyond cancer using previously published Cochrane methods [5].

## Methods

### Search strategy

We performed this review using methods from previous Cochrane systematic reviews by our group [5, 35]. In addition, we supplemented this approach with criterion to identify remotely delivered exercise intervention. Further, due to early and evolving nature of the field, we included studies with mixed cancer cohorts. Searches were updated to November 2020.

Full text articles which had been included in the previous two reviews and those articles that were excluded solely because they included a mixed cancer cohort were screened for inclusion. The searches were run using the same search strategy for the original review from inception to August 2012 [35] and the updated review from August 2012 to May 2018 [5]. Full search strategies can be found in the Supplementary materials. The subsequent searches from the following electronic databases were run from May 2018 up to November 2020: Cochrane Central Register of Controlled Trials, MEDLINE, EMBASE, AMED, CINAHL, PsychINFO, Sport DISCUS, Pubmed central and PEDro. We imported results from each database into a reference management software package (Refworks, Proquest, Michigan, USA), from which we removed duplicates.

### Eligible studies

Randomised controlled trials (RCTs) that were aimed at promoting aerobic and/or resistance exercise behaviours in adults (> 18 years of age) who were physically inactive or had a sedentary lifestyle at baseline (i.e. engaging in less than 30 min of exercise of at least moderate intensity, 3 days per week, or less than 90 min in total of moderate intensity exercise per week) were included. Participants must have been diagnosed with cancer (histologically or clinically) irrespective of sex, tumour type, tumour site, tumour stage and type of anticancer treatment received. Only studies that took place during or after primary treatment or during active monitoring were included.

Only interventions delivered remotely were included (i.e. the intervention was delivered without face to face contact or travel to a dedicated facility beyond the first week of the intervention). Studies must include at least 6 weeks of follow-up. Studies must have reported the frequency, duration and intensity of aerobic exercise behaviour or frequency, intensity, type, sets and repetitions of resistance exercise behaviour that was prescribed in the intervention. We excluded studies directed specifically at end-of-life-care patients and individuals who were currently hospital inpatients. We did not include studies of ‘at risk’ populations (i.e. studies involving individuals who have not been diagnosed with cancer) that addressed primary prevention research questions.

### Assessment of risk of bias in included studies

Risk of bias and methodological quality were assessed in accordance with Cochrane’s tool for assessing risk of bias [36]. We did not include judgements of the risk of bias caused by blinding to group allocation, as it is not possible (e.g. in a supervised exercise setting) to blind participants to an intervention while promoting exercise behaviour.

Two review authors (SI and LB) independently applied the ‘Risk of bias’ tool, and differences were resolved by discussion with a third review author (RT). We summarised results in both a ‘Risk of bias’ graph (Fig. 3) and a ‘Risk of bias’ summary (Fig. 2). We contacted study authors to ask for additional information or for further clarification of study methods if any doubt surrounded potential sources of bias.

### Outcomes

The primary outcomes were aerobic exercise behaviour (frequency, duration, and intensity) and resistance exercise behaviour (sets, repetitions, and intensity). Interventions were judged as successfully achieving exercise goals if investigators reported at least 75% adherence over a given follow-up period [37] as per previous reviews [5, 35].

We also assessed which interventions prescribed aerobic exercise of at least 150 min per week and with at least 2 days per week of strength training, in line with the American Cancer Society’s guidelines for exercise in people living with and beyond cancer [6]. Our secondary outcomes were change in aerobic fitness or exercise tolerance, change in skeletal muscle strength and endurance, adverse events, study recruitment rate and intervention attrition rate.

### Screening and data extraction

Titles and abstracts were interrogated for eligibility by a single reviewer (SI). Full text versions of potentially eligible articles were then independently screened against the inclusion criteria by two reviewers (SI and LB). Disagreements between reviewers were resolved by discussion with other members of the research team (RT). We contacted study authors if we could not access a full text, if we required more information to determine whether a study could be included, or if we required additional information about an already eligible study.

### Data synthesis

We quantified data regarding adherence to the intervention in terms of number of prescribed exercise sessions completed as a proportion of the total prescription. The ‘Coventry, Aberdeen & London—Refined’ (CALO-RE) taxonomy [38] was used to code behaviour-change techniques (BCTs) within the interventions. Where appropriate, meta-analyses of review outcomes were performed. A fixed-effect model was used if there was no significant statistical heterogeneity (I^2^ ≥ 50%). For continuous outcomes, the standardised mean difference between treatment arms was estimated by extracting the final value and standard deviation of the outcome of interest with the number of participants assessed at follow-up. The Cochrane group RevMan version 5.3 (Review Manager 2014) software was used to carry out meta-analyses. If a meta-analysis was not possible or was not appropriate, we narratively synthesised studies.

## Results

**Fig. 1:**
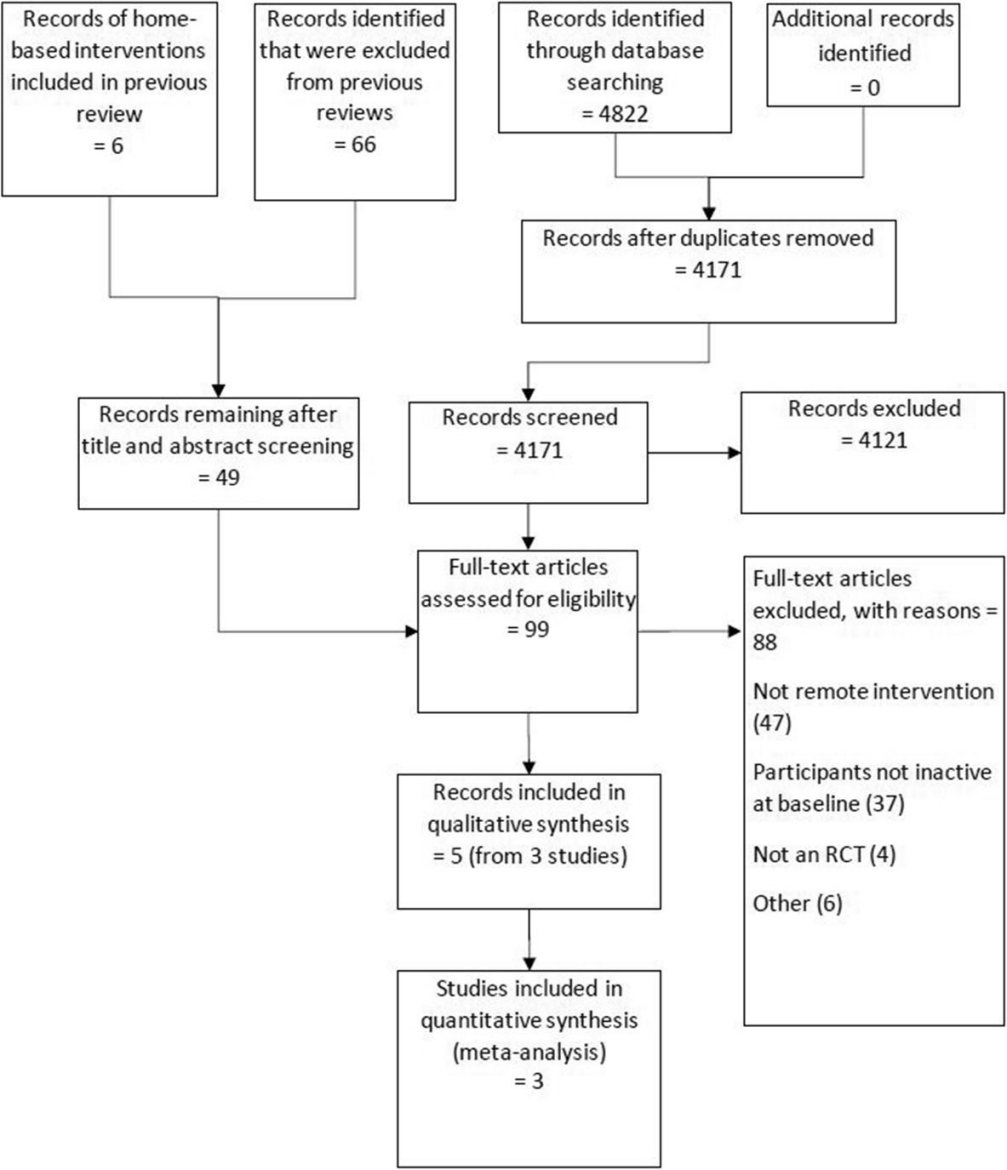
PRISMA flow chart illustrating the study selection process

We identified 4171 unique records from research databases in addition to 12,325 records identified in previous reviews. Seventy-two records were identified from title and abstract screening from previous searches (see Fig. 1, PRISMA flow chart). Including 50 records identified in the updated search, 99 manuscripts were evaluated at the full text stage.

After consensus agreement between study authors, 3 RCTs were included in this review [39–41]. A total of 186 participants were randomised in these trials. A total of 4121 reports were excluded; reasons for exclusion were: non RCTs – for example, reviews or comment/editorial articles; cohorts that included non-cancer populations; trials that were not remote trials that failed to describe essential metrics of exercise prescription used in the intervention; trials involving active participants at baseline; trials involving hospital inpatients; interventions that were < 6 weeks in follow-up; trials involving participants < 18 years of age; not able to translate into English.

Two trials [39, 41] included exclusively female breast cancer survivors whilst Pinto et al. 2013 [40] included both male and female survivors of colorectal cancer. All trials included participants who had completed primary or adjuvant therapy though one trial included participants who were receiving hormone-based therapy [41]. No studies included participants with metastatic disease. Two trials included predominantly/overwhelmingly Caucasian (Pinto et al. 2005[39]- 95%; Pinto et al. 2013[40]- 97%) with one trial not reporting the ethnicity of participants. All trials took place in the North-eastern states of the USA. Comorbidities at baseline were largely unclear or unreported.

Two trials [39, 40] exclusively prescribed aerobic exercise which could take the form of brisk walking, cycling, swimming or using home exercise equipment though it is unclear what activities were performed. One trial [41] allocated individuals to perform aerobic exercise (walking), resistance exercise (resistance band exercises), both or flexibility training (control). Two trials [39, 40] explicitly asked control participants not to modify their existing exercise behaviours. One trial [41] provided participants with written guidance regarding how to exercise at home. In all trials, contact was limited to weekly phone calls, or weekly phone or email contacts [41], after an initial one-to-one exercise consultation with research staff. It was unclear whether healthcare professionals played a role in the interventions [39–41]. All trials delivered their interventions over an initial 12-week period. One trial [39] followed-up participants again at 6- and 9-months post-randomisation and another [40] followed-up participants at 6- and 12-months post-randomisation, with both trials providing monthly phone calls for 3 months after the initial 12-week intervention period. In line with the recommendations of Rock et al. [6]: one study [40] prescribed exercise that complied with the recommendation to perform at least 150 min of aerobic exercise of per week and one study [41] complied with the recommendation to prescribe resistance exercise on at least 2 days per week.

All interventions were explicitly based on a theoretical model. One intervention was based on the exercise and self-esteem theory [41, 42], one on the transtheoretical model alone [39, 43] and one combined the social cognitive theory and transthoretical model [40, 43, 44]. It is unclear whether the prescription of flexibility exercise to the control arm of the trial of Musanti [41] may have influenced the self-esteem of the control group. Details of the BCTs used in each of the included studies are presented in Table 1. Full details of intervention behaviour change technique (BCT) coding according to the CALO-RE taxonomy [38] can be found in the previous review [35].

**Table 1:** Characteristics of included studies

Author & year	Participants	Intervention	Exercise frequency	Exercise intensity	Measurement of exercise behaviour	Adherence	Behaviour change techniques	Theoretical basis
Musanti 2012 [41]	55 women with Stage I–IIIB breast cancer Age: 50.5 ± 7.5 years	12 weeks of home-based exercise with written guidance, self-monitoring of heart rate and weekly phone calls and an exercise log	Aerobic exercise (3–5 bouts of 15–30 min per week) or Resistance exercise (3 bouts of 10–12 reps of 8 exercises for 1 set per week) or Aerobic and resistance (3–5 bouts of aerobic and 2 bouts of resistance per week)	Aerobic exercise: 40–65% of the estimated maximal heart rate Resistance exercise: 3–6/10 on Borg CR 10 scale, resistance was progressed if and RPE of > 6/10 was achieved by the 12th repetition.	Exercise logs completed by participants	Unclear; 81–91% adherence to the exercise prescription was reported at 12 weeks but only 50% of the participants completed exercise logs.	9. Graded tasks, 16. Prompt self-monitoring of behaviour, 17. Prompt self-monitoring of behavioural outcome, 21. Instruction on how to perform behaviour, 22. Demonstration of behaviour, 26. Prompt practice	Exercise and self-esteem model [42]
Pinto et al. 2005 [39]	86 women with Stage 0–II breast cancer Age: 53.1 ± 9.8 years	12 weeks of home-based exercise self-monitoring of activity via heart rate, pedometers and an exercise log with weekly phone calls	Aerobic exercise; 2–5 bouts per weeks for 10–30 min per bout	55–65% of estimated maximum heart rate	7-day Physical activity recall – interviewer administered questionnaire, 3-day uniaxial accelerometry	55.8% of participants meeting prescription of 150 min of physical activity per week at 12 weeks.	5. Goal setting, 8. Barrier identification/Problem solving, 12. Prompt rewards contingent on effort or progress towards goal, 16. Prompt self-monitoring of behaviour, 17. Prompt self-monitoring of behavioural outcome, 19. Feedback on performance	Transtheoretical model [43]
Pinto et al. 2013 [40]	26 women and 20 men with colorectal cancer (26 with colon cancer and 20 with rectal cancer) Age: 57.6 ± 11.2 years	12 weeks of home-based physical activity counselling with self-monitoring of activity via heart rate, pedometers and an exercise log with and weekly phone calls	Aerobic exercise; 2–5 bouts per weeks for 10–30 min per bout	64–76% of estimated maximum heart rate	7-day Physical activity recall – interviewer administered questionnaire, 3-day uniaxial accelerometry, CHAMPS questionnaire	64.7% of individuals achieved 150 min per week of physical activity after 12 weeks vs 40.9% of controls	5. Goal setting, 8. Barrier identification/Problem solving, 9. Graded tasks, 12. Prompt rewards contingent on effort or progress towards goal, 16. Prompt self-monitoring of behaviour, 17. Prompt self-monitoring of behavioural outcome, 19. Feedback on performance, 21. Instruction on how to perform behaviour, 23. Teaching to use prompts, 24. Environmental restructuring, 26. Prompt practice	Transtheoretical model [43], Social cognitive theory [44]

All studies attempted to validate self-reported exercise behaviour with pedometers [41] or uniaxial accelerometers [39, 40], however each study reported conflicting results. Data regarding device-measured physical activity were either not supportive of the self-reported exercise behaviour data [39], were not reported sufficiently to make a judgment [40] or their use was discontinued during the study due to participants having difficulty using them [41]. One study [41] also attempted to use heart rate monitoring via a wearable sensor to objectively verify the exercise intensities achieved by participants but the use of this device was discontinued as participants reported finding it difficult to use.

### Risk of bias in included studies

**Fig. 2:**
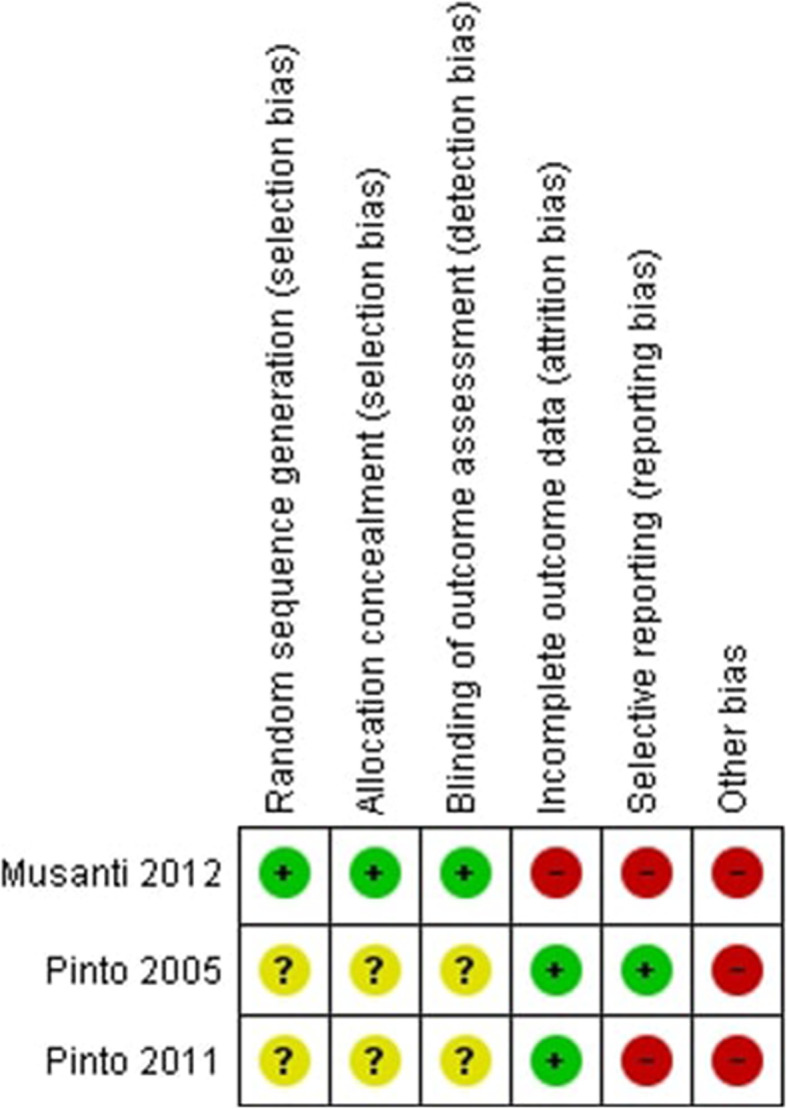
Risk of bias summary: review authors’ judgements about each risk of bias item for each included study

**Fig. 3:**
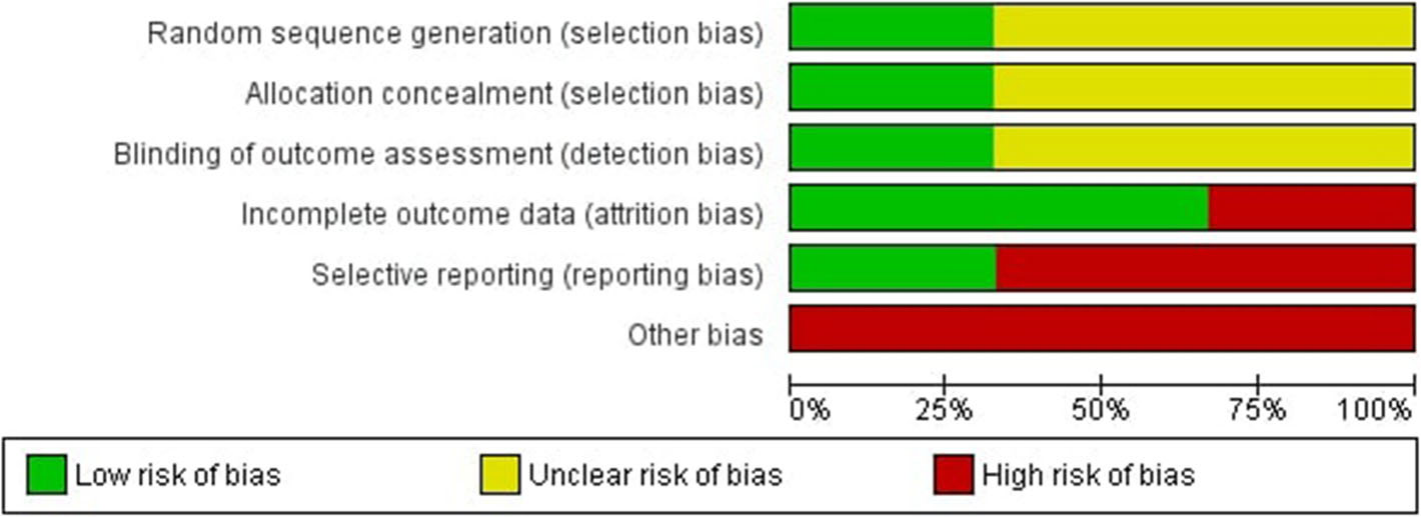
‘Risk of bias’ graph: review authors’ judgements about each risk of bias item presented as percentages across all included studies

All studies included in the review were deemed to have a high risk of bias. Full results of the risk of bias assessment are illustrated in Figs. 2 and 3. One study used an intention to treat analysis [39]. Two studies [39, 40] provided insufficient information to judge the risk related to random sequence generation, allocation concealment or blinding of outcome assessments. One study [41] was judged as having a high risk of attrition bias as 24% (13/54) of participants did not complete their assigned 12-week programmes. Two studies were judged to have a high risk of reporting bias with one [41] failing to report waist and upper, mid and lower arm circumference outcomes and another [40] not reporting accelerometer derived physical activity data.

Other potential sources of bias of note include: missing adherence data for 50% of the participants [41], in study dropouts significantly greater baseline levels of body fat, greater fatigue, greater muscular endurance, lower levels of physical activity and a higher frequency of allocation to resistance training were observed [41], potential differences between cohorts at baseline [39], inconsistencies between device objective/device-based measures and subjective measures of physical activity/exercise behaviour [39, 40].

### Outcomes

No trials reported a 75% adherence to the Rock et al. [6] guidelines for aerobic or resistance exercise recommendations.

**Fig. 4:**
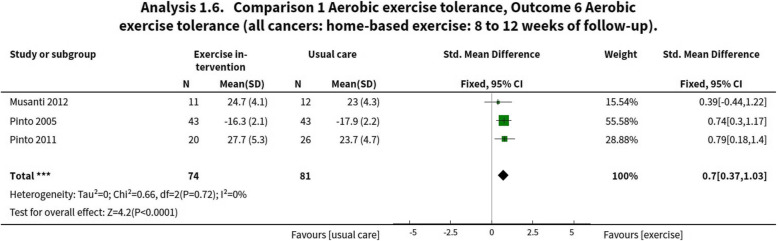
Meta-analysis of aerobic exercise tolerance at 8–12 weeks of follow-up. Note, in all meta-analysis data from Pinto et al. 2005 [39] has been multiplied by − 1 to control for direction of effect (that is, lower values in a timed test indicate a better outcome). Data were extracted from the combined aerobic and resistance training arm of [41]

All studies included a measure of aerobic exercise tolerance which took the form of the Rockport 1-mile walk test [39], a Treadwalk test [40] and a modified Bruce protocol submaximal treadmill test. Aerobic exercise tolerance was unchanged in the intervention versus the control at 12 weeks (SMD 0.70, 95% CI 0.37 to 1.03, 155 participants; very low-certainty evidence – Fig. 4). The certainty of the evidence was graded as very low due to high risk of bias, low number of participants within the studies and wide confidence intervals.

One study reported changes in skeletal muscle strength and endurance [41]. Skeletal muscle strength was assessed as the six-repetition maximum using a chest press, seated row and leg press and was significantly increased at 12 weeks in those who performed resistance training or aerobic and resistance training. Skeletal muscle endurance was assessed using the curl-up test and the YMCA bench press endurance test and significantly increased in the resistance training, aerobic and resistance training, and flexibility training but not the aerobic training group. However, this study suffered from a high risk of bias.

All studies reported adverse events. One study [40] recorded a cancer recurrence in the control arm. Another study [39] reported a participant developing symptoms of chest pain which they refused to have evaluated in the intervention group. Finally [41], reported two cases of recurrence of previous tendinitis: one in the shoulder and the other in the foot; causing participants to temporarily discontinue exercising and adopt lower exercise intensities upon restarting.

Study recruitment rate ranged from 23% [41] to 70% [39, 40]. Two studies used sample size estimates [40, 41] though neither were successful in achieving their recruitment target. Two studies produced CONSORT diagrams [39, 40]. Intervention attrition rates were reported by all studies and ranged from 5% [39] and 7% [40] to 23% [41].

## Discussion

The findings of this review indicate that there is insufficient evidence to conclude that existing remote exercise interventions are useful for achieving the Rock et al. [6] guidelines of 150 min per week of aerobic exercise and twice per week of resistance exercise, in sedentary adult cancer cohorts. The scant availability of evidence was apparent from the review process which highlights the lack of research into the efficacy of remote exercise interventions. Compared to another review which included 29 RCTs [34] we have only included 3 RCTs, owing to stricter inclusion criteria which aimed to identify methodological robust trials. The limited methodological quality of published exercise studies in cancer populations has been noted previously [35]. Key issues that persist are the reporting of exercise prescriptions in a way that can be reproduced and failing to consider the activity status of representative cancer populations and the influence of baseline activity status upon behavioural and physiological outcomes as part of trial design. A simple proposal to improve the reporting of interventions in exercise oncology is for authors to use a research checklist such as the “template for intervention description and replication (TIDieR) checklist” [45] or the “Consensus on exercise reporting template” [46].

Despite the lack of adherence to the exercise prescriptions reported in the included trials, the interventions appeared to cause improvements in aerobic exercise tolerance at 12 weeks of follow-up. This may have resulted from the relatively large early gains in function expected in sedentary participants from exercise training, which at a group level could mask smaller changes in non-adherers. Improvements in aerobic exercise tolerance may not always be indicative of changes in aerobic fitness caused by physiological adaptations to exercise training and may partially reflect familiarization with feelings of exertion during exercise testing and a better tolerance towards perceptions of fatigue. Considering this, alongside the very low quality of evidence for this outcome and small sample size, we conclude that these data are insufficient to support an effect on aerobic exercise tolerance. Similarly, for the other secondary outcomes assessed the limited amount and quality of evidence for changes skeletal muscle strength and endurance, study recruitment rate and intervention attrition rate preclude the drawing of definitive conclusions. There was a high risk of bias for selective reporting, incomplete outcome data and other trial-specific potential biases such as contamination and baseline imbalances. Despite this high risk of bias, the overall conclusions of this review are unchanged.

There were few adverse events identified from the studies included in this review. Two instances of tendonitis recurrence that occurred could potentially be attributed participation in an exercise intervention – though this may result from a detection bias in the intervention arm. The reported case of a cancer recurrence was not unexpected. The definitions of adverse events were not prespecified and as such it is unclear whether low-grade adverse events (e.g. hot flushes, fatigue, nausea, joint pain) which are typical in these populations were recorded. Information about low the prevalence of low-grade adverse events would be useful in further elucidating the role of exercise in cancer care.

The recording and reporting of physical activity was incomplete in the included studies which limits our understanding of the fidelity of their interventions. Self-reported measures of physical activity could not be verified with device-based measures of activity for several reasons including measurement error [47] and poor adherence to wearing activity monitors such as accelerometers or heart rate monitors. Heart rate monitors and accelerometers can provide important data about intervention fidelity and can potentially contribute to intervention adherence by facilitating self-monitoring behaviour. It is important to report barriers to the use of such technologies and understand the effect that the use of such devices may have on intervention outcomes to guide future research.

A pertinent finding from this review is the lack of high-quality trials with reproducible interventions investigating the efficacy of remote exercise interventions in cancer populations. A possible reason for this is the relative infancy of cancer rehabilitation as a field compared to better established clinical exercise services such as cardiac rehabilitation [48]. Previous work in this area has rightly focussed on establishing the efficacy of exercise as a therapy which has typically been conducted in highly controlled research settings with participants being closely supervised. The next steps for this field are to establish how best to promote the exercise behaviours that induce the desired physiological effect and resulting clinical outcomes - the topic of two Cochrane systematic reviews [5, 35].

Although all studies included in this review employed BCTs and had a theoretical basis for the promotion of exercise behaviours, no study achieved acceptable adherence. In contrast, the use of BCTs was a common feature of exercise interventions with better adherence in our previous review [5]. However, there are uncertainties surrounding how well BCTs are implemented and reported, how they interact with the context of a remote intervention and with a how this mediates their effects on health behaviours [49] which may explain this incongruity. Trials in this review provided behavioural support using weekly telephone calls, written information and/or activity monitoring; there are many other emerging technologies (e.g. video calling) that can be utilised to provide behavioural support remotely, each of which presents new contexts for the delivery of BCTs, which must be understood and evaluated. Concerns about the lack of engagement by the elderly with technology [50] may have been stifled by the COVID-19 pandemic with an increase in the proportion of people aged 65+ who engage in weekly video calls from 23% in February 2020 to 61% in April/May 2020 [51]. Further research is needed to understand which BCTs and how BCTs may be used most effectively in remote exercise interventions in sedentary cancer populations. This research can then inform the design of adequately powered long term RCTs and develop further understanding of whether such interventions are effective in promoting exercise behaviour in cancer survivors.

### Limitations

Most of the limitations of the findings of this review are due to the very small sample of studies and participants that were identified – which itself is a principle finding. Though this review aimed to address what was known about remote exercise in a broad range of populations the data available was limited to those who were largely female, Caucasian and based in North Eastern America. This narrow sample of research participants combined with the imprecise estimates of treatment effects that result from the small total sample size leaves substantial uncertainty about the transportability of the findings of this review into other healthcare contexts. Clearly, there is a need for further research in more diverse populations and healthcare settings may influence the participants engagement with and responses to interventions. How the exercise prescriptions employed by the studies in included in this review may have influenced any of the outcomes measured is hard to determine due to the limited adherence to the interventions and compromised assessment of activity from wearable devices and self-report measures.

## Conclusion

At present, there is insufficient evidence to confirm whether remote exercise interventions are useful in promoting exercise behaviours and improving physical function in sedentary adults living with and beyond cancer. This is largely due to a lack of studies addressing this issue. The development and evaluation of novel remote exercise interventions is needed to establish whether these are useful tools for clinical practice.

## Supplementary Information

**Additional file 1.**

## Data Availability

Data are available upon from Professor Bourke (L.Bourke@shu.ac.uk) upon reasonable request.

## Abbreviations

BCT: Behaviour change technique
CALO-RE: ‘Coventry, Aberdeen & London—Refined’
CI: Confidence interval
COVID-19: Coronavirus disease 2019
RCT: Randomised control trial
SMD: Standardised mean difference
