# Supplementary material for: Remote interventions to improve exercise behaviour in sedentary people living with and beyond cancer: a systematic review and meta-analysis
Source: BMC Cancer. 2021 Mar 24;21:308. doi: 10.1186/s12885-021-07989-0 (PMC7987748; doi:10.1186/s12885-021-07989-0)
Supplement: Supplementary file 1 — Additional file 1. [file 12885_2021_7989_MOESM1_ESM.docx]

Supplementary material

Search Strategies

CENTRAL search strategy

CENTRAL 2018 and 2020 update search

#1 MeSH descriptor Neoplasms explode all trees

#2 (cancer* or tumor* or tumour* or neoplas* or malignan* or carcinoma* or adenocarcinoma* or choriocarcinoma* or leukemia* or leukaemia* or metastat* or sarcoma* or teratoma*)

#3 (#1 OR #2)

#4 MeSH descriptor Exercise explode all trees

#5 MeSH descriptor Exercise Movement Techniques explode all trees

#6 MeSH descriptor Exercise Therapy explode all trees

#7 MeSH descriptor Physical Fitness, this term only

#8 (physical* adj5 (fit* or activ*))

#9 (exercis* or aerobic* or resistance* or strength* or walk* or endurance* or lifestyle* or behav*)

#10 (#4 OR #5 OR #6 OR #7 OR #8 OR #9)

#11 #3 and #10

#12 MeSH descriptor: [Health Behavior] explode all trees

#13 MeSH descriptor: [Risk Reduction Behavior] this term only

#14 ((promot* or motivat* or advis* or encourag* or assist* or develop* or stimulat* or help* or support* or organis* or aid* or assist* or endors* or prompt* or driv* or inspire* or lead* or inspir* or further* or advocat* or recommend* or endorse* or foster* or champion*) near/5 (exercis* or aerobic* or resistance* or strength* or walk* or endurance*))

#15 #12 or #13 or #14

#16 #11 and #15

CENTRAL 2012 search

#1 MeSH descriptor Neoplasms explode all trees

#2 (cancer* or tumor* or tumour* or neoplas* or malignan* or carcinoma* or adenocarcinoma* or choriocarcinoma* or leukemia* or leukaemia* or metastat* or sarcoma* or teratoma*)

#3 (#1 OR #2)

#4 MeSH descriptor Exercise explode all trees

#5 MeSH descriptor Exercise Movement Techniques explode all trees

#6 MeSH descriptor Exercise Therapy explode all trees

#7 MeSH descriptor Physical Fitness, this term only

#8 (physical* adj5 (fit* or activ*))

#9 (exercis* or aerobic* or resistance* or strength* or walk* or endurance*)

#10 (#4 OR #5 OR #6 OR #7 OR #8 OR #9)

#11 MeSH descriptor Patient Education as Topic, this term only

#12 (educat* or inform* or teach* or supervis* or communicat* or leaflet*)

#13 MeSH descriptor Survivors, this term only

#14 survivor*

#15 MeSH descriptor Behavior Therapy explode all trees

#16 (behaviour* or behavior* or cognit* or CBT)

#17 MeSH descriptor Motivation explode all trees

#18 MeSH descriptor Interview, Psychological, this term only

#19 (motivat* or interview*)

#20 (#11 OR #12 OR #13 OR #14 OR #15 OR #16 OR #17 OR #18 OR #19)

#21 (#3 AND #10 AND #20)

MEDLINE search strategy

MEDLINE 2018 and 2020 update search

1. exp Neoplasms/

2. (cancer* or tumor* or tumour* or neoplas* or malignan* or carcinoma* or adenocarcinoma* or choriocarcinoma* or leukemia* or leukaemia* or metastat* or sarcoma* or teratoma*).ti,ab.

3. 1 or 2

4. exp Exercise/

5. exp Exercise Movement Techniques/

6. exp Exercise Therapy/

7. Physical Fitness/

8. (physical* adj5 (fit* or activ*)).ti,ab.

9. (exercis* or aerobic* or resistance* or strength* or walk* or endurance* or lifestyle* or behave*).mp.

10. 4 or 5 or 6 or 7 or 8 or 9

11. 3 and 10

12. exp Health Behavior/

13. risk reduction behavior/

14. ((promot* or motivat* or advis* or encourag* or assist* or develop* or stimulat* or help* or support* or organis* or aid* or assist* or endors* or prompt* or driv* or inspire* or lead* or inspir* or further* or advocat* or recommend* or endorse* or foster* or champion*) adj5 (exercis* or aerobic* or resistance* or strength* or walk* or endurance*)).ti,ab.

15. 12 or 13 or 14

16. 11 and 15

17. randomized controlled trial.pt.

18. controlled clinical trial.pt.

19. randomized.ab.

20. placebo.ab.

21. clinical trials as topic.sh.

22. randomly.ab.

23. trial.ti.

24. 17 or 18 or 19 or 20 or 21 or 22 or 23

25. (animals not (humans and animals)).sh.

26. 24 not 25

27. 16 and 26

key:

mp=title, abstract, original title, name of substance word, subject heading word, protocol supplementary concept, rare disease supplementary concept, unique identifier

pt=publication type

ab=abstract

ti=title

sh=subject heading

MEDLINE 2012 search

1. exp Neoplasms/

2. (cancer* or tumor* or tumour* or neoplas* or malignan* or carcinoma* or adenocarcinoma* or choriocarcinoma* or leukemia* or leukaemia* or metastat* or sarcoma* or teratoma*).mp.

3. 1 or 2

4. exp Exercise/

5. exp Exercise Movement Techniques/

6. exp Exercise Therapy/

7. Physical Fitness/

8. (physical* adj5 (fit* or activ*)).mp.

9. (exercis* or aerobic* or resistance* or strength* or walk* or endurance*).mp.

10. 4 or 5 or 6 or 7 or 8 or 9

11. Patient Education as Topic/

12. Patient education handout/

13. (educat* or inform* or teach* or supervis* or communicat* or leaflet*).mp.

14. Survivors/ or survivor*.mp.

15. exp Behavior Therapy/

16. (behaviour* or behavior* or cognit* or CBT).mp.

17. exp Motivation/

18. Interview, Psychological/

19. (motivat* or interview*).mp.

20. 11 or 12 or 13 or 14 or 15 or 16 or 17 or 18 or 19

21. 3 and 10 and 20

22. randomized controlled trial.pt.

23. controlled clinical trial.pt.

24. randomized.ab.

25. placebo.ab.

26. clinical trials as topic.sh.

27. randomly.ab.

28. trial.ti.

29. 22 or 23 or 24 or 25 or 26 or 27 or 28

30. 21 and 29

31. exp animals/ not humans.sh.

32. 30 not 31

key:

mp=title, abstract, original title, name of substance word, subject heading word, protocol supplementary concept, rare disease supplementary concept, unique identifier

pt=publication type

ab=abstract

ti=title

sh=subject heading

Embase search strategy

Embase 2018 and 2020 update search

1. exp neoplasm/

2. (cancer* or tumor* or tumour* or neoplas* or malignan* or carcinoma* or adenocarcinoma* or choriocarcinoma* or leukemia* or leukaemia* or metastat* or sarcoma* or teratoma*).ti,ab.

3. 1 or 2

4. exp exercise/

5. exp kinesiotherapy/

6. fitness/

7. (physical* adj5 (fit* or activ*)).ti,ab.

8. (exercis* or aerobic* or resistance* or strength* or walk* or endurance* or lifestyle* or behav*).mp.

9. 4 or 5 or 6 or 7 or 8

10. 3 and 9

11. exp health behavior/

12. risk reduction/

13. ((promot* or motivat* or advis* or encourag* or assist* or develop* or stimulat* or help* or support* or organis* or aid* or assist* or endors* or prompt* or driv* or inspire* or lead* or inspir* or further* or advocat* or recommend* or endorse* or foster* or champion*) adj5 (exercis* or aerobic* or resistance* or strength* or walk* or endurance*)).ti,ab.

14. 11 or 12 or 13

15. 10 and 14

16. crossover procedure/

17. double‐blind procedure/

18. randomized controlled trial/

19. single‐blind procedure/

20. random*.mp.

21. factorial*.mp.

22. (crossover* or cross over* or cross‐over*).mp.

23. placebo*.mp.

24. (double* adj blind*).mp.

25. (singl* adj blind*).mp.

26. assign*.mp.

27. allocat*.mp.

28. volunteer*.mp.

29. 16 or 17 or 18 or 19 or 20 or 21 or 22 or 23 or 24 or 25 or 26 or 27 or 28

30. 15 and 29

31. (exp animal/ or nonhuman/ or exp animal experiment/) not human/

32. 30 not 31

key:

[mp=title, abstract, subject headings, heading word, drug trade name, original title, device manufacturer, drug manufacturer, device trade name, keyword]

Embase 2012 search

1 exp neoplasm/

2 (cancer* or tumor* or tumour* or neoplas* or malignan* or carcinoma* or adenocarcinoma* or choriocarcinoma* or leukemia* or leukaemia* or metastat* or sarcoma* or teratoma*).mp.

3 1 or 2

4 exp exercise/

5 exp kinesiotherapy/

6 fitness/

7 (physical* adj5 (fit* or activ*)).mp.

8 (exercis* or aerobic* or resistance* or strength* or walk* or endurance*).mp.

9 4 or 5 or 6 or 7 or 8

10 patient education/

11 (educat* or inform* or teach* or supervis* or communicat* or leaflet*).mp.

12 survivor/ or survivor*.mp.

13 behavior therapy/

14 cognitive therapy/

15 (behaviour* or behavior* or cognit* or CBT).mp.

16 motivation/

17 interview/

18 (motivat* or interview*).mp.

19 10 or 11 or 12 or 13 or 14 or 15 or 16 or 17 or 18

20 3 and 9 and 19

21 crossover procedure/

22 double‐blind procedure/

23 randomized controlled trial/

24 single‐blind procedure/

25 random*.mp.

26 factorial*.mp.

27 (crossover* or cross over* or cross‐over*).mp.

28 placebo*.mp.

29 (double* adj blind*).mp.

30 (singl* adj blind*).mp.

31 assign*.mp.

32 allocat*.mp.

33 volunteer*.mp.

34 21 or 22 or 23 or 24 or 25 or 26 or 27 or 28 or 29 or 30 or 31 or 32 or 33

35 20 and 34

36 (exp animal/ or nonhuman/ or exp animal experiment/) not human/

37 35 not 36

key:

[mp=title, abstract, subject headings, heading word, drug trade name, original title, device manufacturer, drug manufacturer, device trade name, keyword]

AMED search strategy

AMED Ovid 2018 and 2020 update search

1 exp neoplasms/

2 (cancer* or tumor* or tumour* or neoplas* or malignan* or carcinoma* or adenocarcinoma* or choriocarcinoma* or leukemia* or leukaemia* or metastat* or sarcoma* or teratoma*).mp.

3 1 or 2

4 exp exercise/

5 exp exercise therapy/

6 physical fitness/

7 (physical* adj5 (fit* or activ*)).mp.

8 (exercis* or aerobic* or resistance* or strength* or walk* or endurance* or lifestyle* or behav*).mp.

9 4 or 5 or 6 or 7 or 8

10 exp Health behavior/

11 ((promot* or motivat* or advis* or encourag* or assist* or develop* or stimulat* or help* or support* or organis* or aid* or assist* or endors* or prompt* or driv* or inspire* or lead* or inspir* or further* or advocat* or recommend* or endorse* or foster* or champion*) adj5 (exercis* or aerobic* or resistance* or strength* or walk* or endurance*)).ti,ab.

12 10 or 11

13 3 and 9 and 12

key:

mp=abstract, heading words, title

AMED Ovid 2012 search

1 exp neoplasms/

2 (cancer* or tumor* or tumour* or neoplas* or malignan* or carcinoma* or adenocarcinoma* or choriocarcinoma* or leukemia* or leukaemia* or metastat* or sarcoma* or teratoma*).mp.

3 1 or 2

4 exp exercise/

5 exp exercise therapy/

6 physical fitness/

7 (physical* adj5 (fit* or activ*)).mp.

8 (exercis* or aerobic* or resistance* or strength* or walk* or endurance*).mp.

9 4 or 5 or 6 or 7 or 8

10 exp patient education/

11 (educat* or inform* or teach* or supervis* or communicat* or leaflet*).mp.

12 survivors/ or survivor*.mp.

13 exp behavior therapy/

14 (behaviour* or behavior* or cognit* or CBT).mp.

15 exp motivation/

16 interviews/

17 (motivat* or interview*).mp.

18 10 or 11 or 12 or 13 or 14 or 15 or 16 or 17

19 3 and 9 and 18

key:

mp=abstract, heading words, title

CINAHL search strategy

CINAHL 2018 and 2020 update search

1 exp NEOPLASMS/

2 (cancer* OR tumor* OR tumour* OR neoplas* OR malignan* OR carcinoma* OR adenocarcinoma* OR choriocarcinoma* OR leukemia* OR leukaemia* OR metastat* OR sarcoma* OR teratoma*).af

3 1 OR 2

4 exp EXERCISE/

5 exp THERAPEUTIC EXERCISE/

6 exp PHYSICAL FITNESS/

7 (physical* AND (fit* OR activ*)).af

8 (exercis* OR aerobic* OR resistance* OR strength* OR walk* OR endurance* or lifestyle* or behave*).af

9 4 OR 5 OR 6 OR 7 OR 8

10 3 and 9

11 exp BEHAVIOR THERAPY/

12. (risk reduction*) AND (behav*)

13 ((promot* or motivat* or advis* or encourag* or assist* or develop* or stimulat* or help* or support* or organis* or aid* or assist* or endors* or prompt* or driv* or inspire* or lead* or inspir* or further* or advocat* or recommend* or endorse* or foster* or champion*) adj5 (exercis* or aerobic* or resistance* or strength* or walk* or endurance*)).ti,ab.

14 11 or 12 or 13

15 10 AND 14

16 Randomized controlled trials

17 Randomised controlled trials

18 16 or 17

19 15 AND 18

key

af=any field

CINAHL 2012 search

1 exp NEOPLASMS/

2 (cancer* OR tumor* OR tumour* OR neoplas* OR malignan* OR carcinoma* OR adenocarcinoma* OR choriocarcinoma* OR leukemia* OR leukaemia* OR metastat* OR sarcoma* OR teratoma*).af

3 1 OR 2

4 exp EXERCISE/

5 exp THERAPEUTIC EXERCISE/

6 exp PHYSICAL FITNESS/

7 (physical* AND (fit* OR activ*)).af

8 (exercis* OR aerobic* OR resistance* OR strength* OR walk* OR endurance*).af

9 4 OR 5 OR 6 OR 7 OR 8

10 exp PATIENT EDUCATION/

11 (educat* OR inform* OR teach* OR supervis* OR communicat* OR leaflet*).af

12 CANCER SURVIVORS/

13 survivor*.af

14 exp BEHAVIOR THERAPY/

15 (behaviour* OR behavior* OR cognit* OR CBT).af

16 exp MOTIVATION/

17 MOTIVATIONAL INTERVIEWING/

18 (motivat* OR interview*).af

19 10 OR 11 OR 12 OR 13 OR 14 OR 15 OR 16 OR 17 OR 18

20 3 AND 9 AND 19

21 RANDOMIZED CONTROLLED TRIALS/

22 20 and 21

PsycINFO search strategy

PsycINFO 2018 and 2020 update search

1 neoplasms.af

2 ((cancer* OR tumor* OR tumour* OR neoplas* OR malignan* OR carcinoma* OR adenocarcinoma* OR choriocarcinoma* OR leukemia* OR leukaemia* OR metastat* OR sarcoma* OR teratoma*)).ti,ab

3 exercise.af

4 (physical AND fitness).af

5 ((physical* adj5 (fit* OR activ*))).ti,ab

6 ((exercis* OR aerobic* OR resistance* OR strength* OR walk* OR endurance* OR lifestyle* OR behave*)).af

7 1 OR 2

8 3 OR 4 OR 5 OR 6

9 (health AND behaviour).af

10 (risk AND reduction AND behaviour).af

11 (((promot* OR motivat* OR advis* OR encourag* OR assist* OR develop* OR stimulat* OR help* OR support* OR organis* OR aid* OR assist* OR endors* OR prompt* OR driv* OR inspire* OR lead* OR inspir* OR further* OR advocat* OR recommend* OR endorse* OR foster* OR champion*) adj5 (exercis* OR aerobic* OR resistance* OR strength* OR walk* OR endurance*))).ti,ab

12 9 OR 10 OR 11

13 7 AND 8 AND 12

PsycINFO Ovid 2012 search

1 exp neoplasms/

2 (cancer* or tumor* or tumour* or neoplas* or malignan* or carcinoma* or adenocarcinoma* or choriocarcinoma* or leukemia* or leukaemia* or metastat* or sarcoma* or teratoma*).mp.

3 1 or 2

4 exp exercise/

5 physical fitness/

6 (physical* adj5 (fit* or activ*)).mp.

7 (exercis* or aerobic* or resistance* or strength* or walk* or endurance*).mp.

8 4 or 5 or 6 or 7

9 client education/

10 (educat* or inform* or teach* or supervis* or communicat* or leaflet*).mp.

11 survivors/ or survivor*.mp.

12 exp cognitive behavior therapy/

13 exp behavior therapy/

14 (behaviour* or behavior* or cognit* or CBT).mp.

15 exp motivation/

16 motivational interviewing/

17 (motivat* or interview*).mp.

18 9 or 10 or 11 or 12 or 13 or 14 or 15 or 16 or 17

19 3 and 8 and 18

20 clinical trials/

21 (random* or trial* or group* or placebo*).mp. mp=title, abstract, heading word, table of contents, key concepts, original title, tests & measures

22 20 or 21

23 19 and 22

key:

[mp=title, abstract, heading word, table of contents, key concepts, original title, tests & measures]

SPORTS DISCUS search strategy (EBSCO host)

Sports discus 2018 and 2020 update search

1. TX cancer* OR tumor* OR tumour* OR neoplas* OR malignan* OR carcinoma* OR adenocarcinoma* OR choriocarcinoma* OR leukemia* OR leukaemia* OR metastat* OR sarcoma* OR teratoma*

2. TX randomi*ed controlled trial

3. (TX randomi*ed controlled trial) AND (S4 AND S5)

4. Limiters ‐ Published Date: 20120101‐20171231

(20171231-20201120 used in 2020 update)

PEDro search strategy

PEDro 2012, 2018 and 2020 search

Title and abstract: “cancer”

Therapy: fitness training (selected)

Sub discipline: oncology (selected)

Method: clinical trial (selected)
